# Cyclodextrin-Based Nanosponges as Perse Antimicrobial Agents Increase the Activity of Natural Antimicrobial Peptide Nisin

**DOI:** 10.3390/pharmaceutics14030685

**Published:** 2022-03-21

**Authors:** Yousef Khazaei Monfared, Mohammad Mahmoudian, Gjylije Hoti, Fabrizio Caldera, José Manuel López Nicolás, Parvin Zakeri-Milani, Adrián Matencio, Francesco Trotta

**Affiliations:** 1 Dipartimento Di Chimica, Università di Torino, Via P. Giuria 7, 10125 Torino, Italy; yousef.khazaeimonfared@unito.it (Y.K.M.); gjylije.hoti@unito.it (G.H.); fabrizio.caldera@unito.it (F.C.); 2 Faculty of Pharmacy, Tabriz University of Medical Sciences, Tabriz 5166414766, Iran; mahmoudian.m@tbzmed.ac.ir; 3 Unidad Docente de Biología, Departamento de Bioquímica y Biología Molecular A, Facultad de Veterinaria, Regional Campus of International Excellence “Campus Mare Nostrum”, Universidad de Murcia, 30100 Murcia, Spain; josemln@um.es; 4 Liver and Gastrointestinal Diseases Research Centre, Faculty of Pharmacy, Tabriz University of Medical Sciences, Tabriz 5166414766, Iran

**Keywords:** cyclodextrin, pyromellitic dianhydride, carbonyldiimidazole, nanosponges, *Staphylococcus aureus*, *Escherichia coli*, antibacterial, nisin Z

## Abstract

At present, antibiotic resistance is considered a real problem. Therefore, for decades scientists have been looking for novel strategies to treat bacterial infections. Nisin Z, an antimicrobial peptide (AMP), can be considered an option, but its usage is mainly limited by the poor stability and short duration of its antimicrobial activity. In this context, cyclodextrin (CD)-based nanosponges (NSs), synthesized using carbonyldiimidazole (CDI) and pyromellitic dianhydride (PMDA), were chosen for nisin Z loading. To determine the minimum inhibitory of nisin Z loaded on CD-NS formulations, agar well diffusion plates were used. Then, the bactericide concentrations of nisin Z loaded on CD-NS formulations were determined against Gram-positive (*Staphylococcus aureus*) and -negative (*Escherichia coli*) bacteria, using microdilution brain heart infusion (BHI) and tetrazolium salt 3-(4,5-dimethylthiazol-2-yl)-2,5-diphenyl tetrazolium bromide (MTT). The minimum and bactericide inhibitory values of the nisin complex with NSs were potentially decreased against both bacteria, compared with the nisin-free sample, while the nisin complex with β-CD showed lower antibacterial activity. The antimicrobial effect was also demonstrated by free NSs. Furthermore, the total viable counts (TVCs) antibacterial experiment indicated that the combination of nisin Z in both PMDA and CDI β-CD-based NSs, especially CDI, can provide a better conservative effect on cooked chicken meat. Generally, the present study outcomes suggest that the cross-linked β-CD-based NSs can present their own antimicrobial potency or serve as promising carriers to deliver and enhance the antibacterial action of nisin Z.

## 1. Introduction

Management of bacterial infections has emerged as a phenomenon that weakens our ability to fight them because they constantly develop a new set of mechanisms that enable them to resist the inhibitory action of antibiotics [1]. The main mechanism of antibiotics inhibition against microbes growth is related to the prevention of the protein or cell wall synthesis in case of infectious conditions, but the most visible limitation for well-established therapeutic antibiotics is related to bacteria resistance, in addition to their inefficient delivery at the specific site [2]. Therefore, the most recommended approach implies higher doses or combinational drug therapy to increase drug efficiency [3,4], which sometimes present adverse effects. It is clear that the timing of antibiotic resistance is important, and addressing how to better slow drug resistance requires an interdisciplinary approach. The traditional medical subject boundaries no longer act as barriers to sharing ideas and concepts in the broad field of biology. Novel strategies, such as the use of novel excipients with intrinsic antimicrobial activity, provide a preferable and strategic alternative to finding new therapeutic approaches that escape or prevent resistance.

For example, one of the most commonly used antimicrobial peptides (AMPs) in the food industry is nisin Z, which is made by *Lactococcus lactis* strains and contains 34 amino acids [5]. It has a 3.5 kDa molecular weight, with a broad spectrum of antibacterial activity against Gram-positive bacteria. Owing to its low level of toxicity for humans, nisin Z has been considered as a “generally recognized as safe” (GRAS) peptide in the food industry by the FAO/WHO [6,7]. The main mechanisms of nisin Z to kill bacteria are based on the formation of pores in the membrane, and the inhibition of cell wall biosynthesis by binding to lipid II, a part of the membrane-anchored cell wall of bacteria that influences the cell-wall synthesis. This facilitates the transmembrane orientation of nisin [8,9,10]. Therefore, the use of nisin as a popular and natural antimicrobial agent in dairy, fruit juices, meat, and plant products has increased due to its capacity against various types of microorganisms [11]. Nevertheless, the uncontrolled antibacterial function during food storage, sensitivity to environmental stresses, sensitivity to proteolysis, and adverse interactions with food components, are the most common nisin-Z challenges that prevent their potential therapeutical applications [11]. To overcome these challenges, several nano-delivery systems based on proteins, polysaccharides, and lipids have been proposed, allowing the sustained release of nisin Z [7,12,13]. The effect of nanoencapsulation systems to increase the release and bioavailability of targeted compounds has been reported [7,12].

The biopolymers, due to their biodegradability, biocompatibility, and nontoxicity, have attracted considerable attention as a delivery system for nisin Z and other bioactive agents, in food packaging, to decrease unpleasant taste and increase their shelf life [7,12,13,14,15,16]. Among the most common biopolymers that have been used for delivering bioactive agents are cyclodextrins (CDs), because of their low cost and high biocompatibility [17,18,19]. CDs are cyclic oligosaccharides containing glucopyranoside monomeric units linked via α-(1,4)-glycosidic bonds with the central lipophilic cavity and outer hydrophilic surface [20,21]. Their applications in the pharmaceutical and nutraceutical industries, among others, have significantly increased [22,23,24]. Under particular conditions, CDs can react with various cross-linkers such as carbonyl diimidazole (CDI) and pyromellitic dianhydride (PMDA), to synthesize highly cross-linked polymers, known as cyclodextrin-based nanosponges (CD-based NSs) [19]. Natural CDs have been considered as “generally recognized as safe” (GRAS) in food products such as food additives and supplements [25,26]; moreover, CD-based polymers can provide promising food applications [23]. From another point of view, drug-loaded nanoparticles provide new pathways to inhibit bacterial growth [27,28,29]. Meanwhile, recently, the prevention of Gram-positive and -negative bacterial growth [30], and the induction of *Mycobacterium tuberculosis* (Mtb) infection in mice [31] by β-CD-based NSs has been investigated. In addition, β-CD, PEI, and phosphonitrile chloride trimmer (PNC) are assembled to produce antibacterial nanoparticles (Eb-CDNs) that can be utilized as effective agents for wastewater treatment. Interestingly, they have shown remarkable antibacterial activity (99.9%) for eliminating the harmful pathogens (*E. coli* and *S. aureus*) that can be found in wastewater [32].

Our recent study provided evidence for the remarkable anticancer activity of nisin Z loaded on PMDA-NSs and CDI-NSs in comparison to nisin-Z-free forms, acting against colon and breast cancer cell lines [33]. Moreover, as nisin Z is considered an antimicrobial peptide, the antimicrobial activity of nisin-Z-loaded NSs needs to be investigated.

Subsequently, the aim of this study was the evaluation of the antimicrobial activity of nisin-Z-free formulation, as well as nisin loaded on native β-CD and on PMDA and CDI-based NSs. The PMDA- and CDI-based NS formulations can be further considered as promising strategies against bacteria such as *Escherichia coli* (*E. coli*), and *Staphylococcus aureus* (*S. aureus*).

To summarize the above-mentioned statements, the objectives of this study are as follows:

1. The enhancement of the antibacterial effect of nisin Z complexed with CD-based NSs and β-CD;
2. The understanding of the antibacterial activity of CD-NSs and β-CD;
3. The evaluation of the influence of CD-NSs and β-CD for enhancing the conservative effect of nisin Z on cooked chicken meat and preventing the decomposition of nisin in present pepsin.

## 2. Materials and Methods

### 2.1. Materials

Nisin Z (2.5% *w*/*w*) and pepsin were obtained from Sigma Aldrich (Milan, Italy). β-CD was purchased from Cyclolab (Budapest, Hungary). *E. coli* (ATCC 25992) and *S. aureus* (ATCC 25923) were prepared from a Persian collection of bacteria in the Iranian Research Organization for Science and Technology (Tehran, Iran). All other reagents used were of analytical grade.

### 2.2. Methods

#### 2.2.1. Synthesis of β-Cyclodextrin-Based Nanosponges

CD-based NSs were synthesized based on previously published protocols [34,35,36], and a 1:4 molar ratio of the β-CD: PMDA and β-CD: CDI NSs was utilized [20,37].

#### 2.2.2. Preparation of Nisin-Z-Loaded β-CD-Based NSs

Nisin Z, through the freeze-drying method, was loaded on β-CD (5 mM), and CD-based NSs in the calculated amount of nisin Z in a weight ratio of 1:4 (drug:β-CD NS), as previously described in the literature [33].

#### 2.2.3. Fourier Transformed Infrared Study

Nisin-Z-free, β-CD, CD-based NSs, and nisin Z loaded on β-CD-based NSs were analyzed using Fourier transform infrared (FTIR) spectroscopic studies (Bruker, Tensor 27, Germany instrument), in the region of 400–4000 cm^−1^, with a resolution of 4 cm^−1^, to find the existence of interactions between nisin Z and β-CD-NS systems. KBr was mixed with the samples (the ratio of KBr to sample is 100:1); then, the KBr pellet was prepared by applying sufficiently high pressure to a homogeneous mixture of the KBr and sample until the pellet turned transparent.

#### 2.2.4. In Vitro Release Study

In contrast to the procedure in our previous article, in which the release profile of nisin Z was examined at physiological pH [33], in this study, the release was examined at an acidic pH (pH = 4) over 48 h at 37 °C. Then, the nisin-Z content in the buffer solution was determined by the Bradford assay, as previously presented [38].

### 2.3. Antimicrobial Activity Assays

#### 2.3.1. Preparation of Bacterial Strains, Medium, and Cultivation

Gram-negative bacteria (*E. coli*) and Gram-positive bacteria (*S. aureus*) were grown, sub-cultured, and maintained in Mueller–Hinton agar and stored at 4 °C. In this experiment, a single colony of each organism was inoculated into 10 mL of brain heart infusion (BHI) broth, and it was then incubated in an incubator (37 °C and 200 rpm) for 24 h. The optical density of the overnight culture was adjusted to that of a 0.5 McFarland standard (1.5 × 10^8^ colony-forming units (CFU)/mL). Through the dilution of the 0.5 McFarland standard with BHI, the final working concentration (1 × 10^6^ CFU/mL) was obtained. Antibacterial activities of the free-nisin-Z and samples complexed with β-CD and NSs at different concentrations were determined by 2-fold dilution of nisin Z, ranging from 5000 to 625 µg/mL, and a suitable quantity of NSs according to the drug loading; in the case of β-CD, the same quantity as NSs were added. Native CDs and NSs were evaluated using carrier quantity. The formulations were as follows: (i) 625 µg/mL: 625 µg/mL nisin + 2500 µg/mL of NS or β-CD; (ii) 1250 µg/mL: 1250 µg/mL nisin + 5000 µg/mL of Ns or β-CD; (iii) 2500 µg/mL: 2500 µg/mL nisin + 10,000 µg/mL of NSs or β-CD and; (iv) 5000 µg/mL: 5000 µg/mL nisin + 20,000 µg/mL of Ns or β-CD; furthermore, the same quantity of NSs and nisin Z complexed with β-CD were used to evaluate antimicrobial effect. Antimicrobial tests were carried out against *S. aureus* and *E. coli* following the Clinical and Laboratory Standards Institute (CLSI) guidelines as previously described, with some modifications [39].

#### 2.3.2. Antimicrobial Evaluation in Solid Culture Medium

An agar well diffusion plate was used to determine the inhibition zone of the nisin-free sample and the one complexed with formulations in Mueller–Hinton agar (MHA) medium. A 20 mL of the agar medium was added to each Petri dish (95 mm × 15 mm) and allowed to solidify. Then, the mediums were inoculated with 20 µL of brain heart infusion (BHI) broth containing bacterial cultures. Further, 75 μL of antimicrobial agents were added to the holes made by sterile pipette tips, with a diameter of 7.0 mm. The calculation of the diameter of the inhibition zone determined the antimicrobial properties of formulations after 24 h incubation of plates at 37 °C.

#### 2.3.3. Antimicrobial Evaluation in Liquid Culture Medium

A modified microdilution brain heart infusion (BHI) broth test was used to show bacterial growth (Optical Density 600 nm) at different time intervals by using 96-well microplates. To determine the minimal inhibitory concentrations (MICs), a visually modified broth microdilution method [40] with yellow tetrazolium salt 3-(4,5-dimethylthiazol-2-yl)-2,5-diphenyl tetrazolium bromide (MTT) was used [39]. The living and dead cells were considered by the observation of purple- and yellow-colored wells. As regards negative and positive control groups, a medium free of bacteria and a medium consisting of bacteria cells without antibiotics or antimicrobial peptides were used, respectively. MICs were considered as the concentrations of the first yellow wells observed in each treatment after being contacted for 24 h with selected formulations. Furthermore, the minimum bactericidal concentrations (MBCs) were determined by spreading aliquots (50 μL) of each well that illustrated MIC value on MHA plates, which were incubated for 24 h at 37 °C. The MBC of selected formulations for each strain was determined as the lowest concentration at which no colony formation appeared on MHA plates.

#### 2.3.4. Capacity of Bacteria to Use β-CD and NSs as Carbon Sources

Drug-free β-CD and nanosponges with indicated concentrations were mixed as a component of agar medium in a minimal media [41] containing NH_4_Cl as nitrogen source and cultivating both positive and negative bacteria on the surface of agar medium to understand the capacity of bacterial to use the β-CD and nanosponges as carbon source.

#### 2.3.5. Total Viable Counts (TVCs)

To determine the antibacterial effect of the nisin-free sample and the sample complexed with the formulations on the chicken cooked meat, the total viable count (TVC) test was used. The fresh chicken meat was kept at 0 °C for 1 h before use, and then similar pieces weighing about 10 g were obtained. Then, samples were washed with deionized water and cooked in boiling water for 20 min. The total viable counts (TVCs) of cooked chicken samples were determined by the aerobic plate count method as previously reported, with some modifications [42]. Firstly, the antimicrobial solutions were freshly prepared including (C) samples treated with distilled water; (N) samples treated with 0.05% nisin; (N-P) samples treated with 0.05% nisin and 0.15% pepsin; (N-β) samples treated with 0.05% nisin and 0.1% β-CD; (N-β-P) samples treated with 0.05% nisin, 0.1% β-CD, and 0.15% pepsin; (CDI-N) 0.05% nisin and 0.1% CDI-NSs; (CDI-N-P) 0.05% nisin, 0.1% CDI-NS, and 0.15% pepsin; (PMDA-N) 0.05% nisin and 0.1% PMDA-NSs; (PMDA-N-P) 0.05% nisin, 0.1%, PMDA-NS, and 0.15% pepsin. The intervals times—namely, 0, 3, 7, 14, and 30 days—were set following the previous protocol [42]. Plate count agar was evaluated after 24 h of storage at 37 °C for TVC determination, and the logarithms of the number of colony-forming units (CFU/mL) were used to report the results.

### 2.4. Statistical Analysis

Data were analyzed by two-way ANOVA and Dunnett’s multiple comparisons test using GraphPad Prism version 8, and *p* < 0.05 was determined as the significance of differences. The results were expressed as means ± standard deviations of three replications (*n* = 3).

## 3. Results and Discussion

### 3.1. Characterization and In Vitro Release

**Figure 1:**
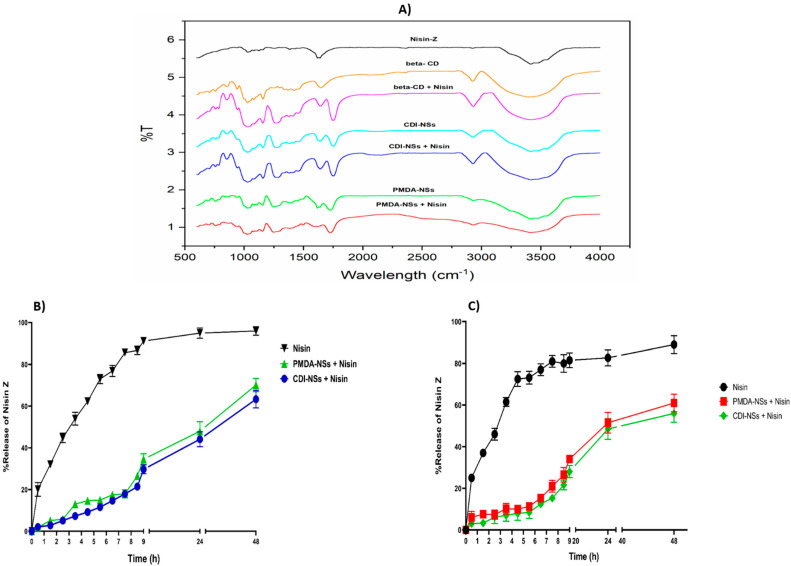
(**A**) FTIR spectra of samples; (**B**) release profiles of nisin and nisin-loaded PMDA/CDI-NSs (dissolution medium: phosphate-buffered solution, pH 4, temperature: 37 ± 0.5 °C, rotation speed: 150 rpm); (**C**) release profiles of nisin and nisin-loaded PMDA/CDI-NSs (dissolution medium: phosphate-buffered solution, pH 7.4, temperature: 37 ± 0.5 °C, rotation speed: 150 rpm; obtained from [33]).

Our previous report [33] demonstrated the possibility of complexing nisin with nanosponges as an effective way to stabilize the peptide. Indeed, good encapsulation efficiency of 90% was found under optimized conditions. In this study, we used the same conditions to prepare the complexes. However, the results of our previous study relatively confirmed the complex formation between nisin Z and both nanosponges by SEM techniques while providing the clearest evidence regarding DLS results and the effect of both NSs to change the release profile of nisin Z [33], as is supported by different publications [43,44]. In this study, a DSC method was carried out to determine the complex formation, with unsuccessful results; for this reason, we carried out FTIR as another validation test, but the results were unclear (Figure 1A). The profiles suggested evidence of complexation in the case of β-CD around 1250–1750, where the shape of the β-CD peaks changed considerably, possibly due to the presence of nisin. On the other hand, the spectra in presence of NSs did not show differences between complex and free NSs, possibly due to the larger structure of NSs. Finally, the antimicrobial use of these complexes needs the characterization of its release properties at acidic pH, where food-related bacteria tend to exist [45]. For this reason, the release profiles were studied at pH 4. The results showed a clear effect of complexation in presence of NSs, with no statistical differences between both NSs. The pH effect was strongly related to the release capacities. Comparing these data with those of pH 7.4 (Figure 1C) [33], we found that at pH 4, the release was slightly higher, even with the nisin-free formulation (Figure 1B). This is because the intrinsic solubility of nisin is higher at acidic pH than that in physiological conditions [46]. These results corroborate and support the idea of the formation of complexes between NS and nisin, as well as their capacity to control release and stability.

### 3.2. Antimicrobial Activity Assays

#### 3.2.1. Inhibition Zone Diameter

**Figure 2:**
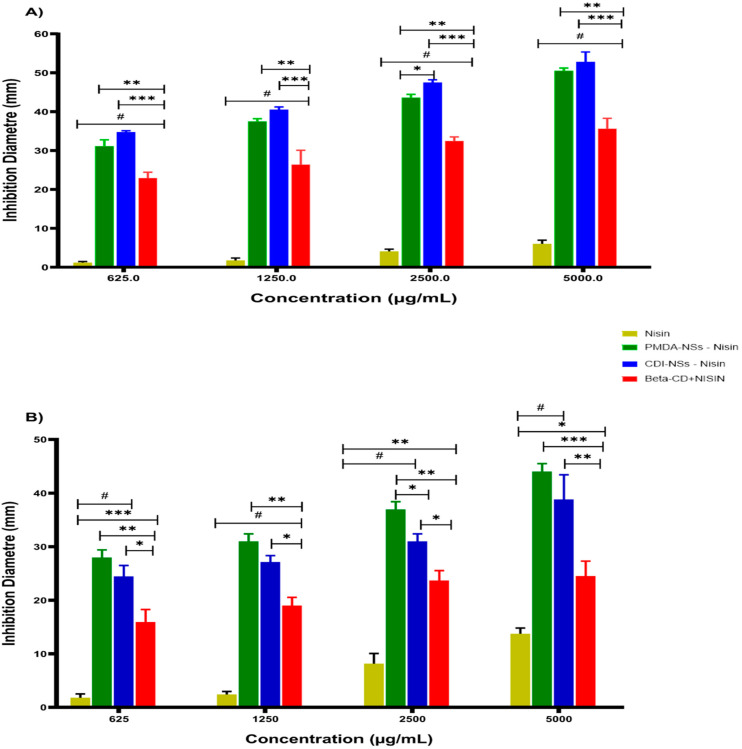
The inhibitory activity (appearance) of nisin Z, PMDA-NSs + nisin Z, CDI-NSs + nisin Z, β-CD + nisin Z. It was evaluated by measuring the growth inhibition zones at different concentrations against *E. coli* (**A**) and *S. aureus* (**B**) in well agar diffusion assay. * *p* < 0.05, ** *p* < 0.01, *** *p* < 0.001, # *p* < 0.0001; ns, not significant. The cited concentrations refer to final nisin concentration; see Section 2.3.1.

Inhibition zones of nisin-free and nisin complexed with PMDA, CDI, and β-CD, and drug-free formulations (Figure S1, Supplementary Materials) against *S. aureus* and *E. coli* were investigated. As shown in Figure 2, the nisin-free sample exhibited a better inhibition zone against *S. aureus* as a Gram-positive bacteria than against *E. coli* as a Gram-negative bacteria, which showed negligible inhibition zone even at the highest concentration, as reported previously [47,48]. As a promising approach in food science in recent years, various studies have revealed the effect of different nanoparticles to improve the inhibition activity of nisin against bacteria, especially Gram-positive bacteria, when compared with nisin-free forms. This fact is in agreement with the results of the present study, which showed that the complex form in PMDA and CDI increased the inhibition zone approximately 10 times more than the nisin-free form at 5000 µg/mL for both strains (*p* < 0.001) [49,50,51,52,53,54]. However, the complex of nisin with β-CD indicated a less inhibitory effect. Perhaps due to the affinity of β-CD with some membrane components [55] because β-CD and the polymers presented some antibacterial capacity, which was higher in the polymeric material. A possible explanation would be the higher number of CD units in the polymer, which would increase the affinity with these molecules, justifying the observed differences. Moreover, the higher entrapment of water into the NS structure might cause a prolonged release of antimicrobial agents such as nisin, leading to a better antibacterial action of nisin in forms complexed with NSs rather than those with β-CD [43].

The results of the present study showed that nisin complexed with CDI-NSs can inhibit the growth of *E. coli* significantly better than the nisin-free form, even at the lowest concentration, which can be attributed to (i) a better formulation that increases the activity of nisin Z [23] or (ii) the antibacterial combinatorial effect of nisin and the nanocarrier, because drug-free CDI-NSs illustrated a notable bacterial inactivation depending on the concentration (Figure S1). These results match with the results of Desai et al., indicating that CDI-NSs with drug-free formulations considerably inhibited the growth of *E. coli* at 5 mg/mL, by about 78.9% [30,56]. This antibacterial effect of CDI-NSs against *E. coli* seems to be associated with the interaction among the outer membrane, lipopolysaccharide, or other biomolecules of the bacterial cell membranes because it is presumably the most sensitive site to be attacked by cyclodextrins and its derivatives [56].

These potential antibacterial effects of nisin entrapped in both NSs and β-CD against *S. aureus* and *E. coli* have been demonstrated with other antimicrobials. For example, allyl isothiocyanate (AIT), a food preservative agent, complexed with β-CD, showed a considerably better antimicrobial effect than unentrapped AIT [57]. Another study showed that the antimicrobial activity of chlorhexidine against Gram-negative bacteria was remarkably enhanced by cyclodextrin encapsulation; indeed, the results of TEM and SEM showed cell membrane structural changes and ultrastructural membrane swelling [58]. Recent data manifested new antibacterial aspects of CDs, specifically α-cyclodextrin, which showed a strong ability to inhibit bacterial activities by changing their communications through blocking the quorum sensing [59].

#### 3.2.2. Inhibition of Bacteria Growth Rate in BHI

**Figure 3:**
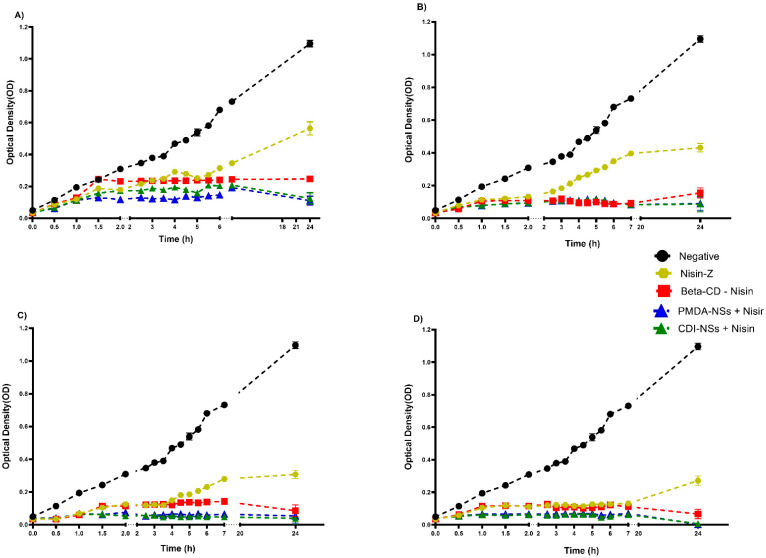
Growth curve of antibacterial activity of nisin-free and complexed in formulation samples against *E. coli*, compared with control and measured by optical density (600 nm) as a function of time: (**A**) 625, (**B**) 1250, (**C**) 2500, and (**D**) 5000 µg/mL. All data are expressed as mean ± standard deviation (*n* = 3). The cited concentrations refer to final nisin concentration; see Section 2.3.1.

**Figure 4:**
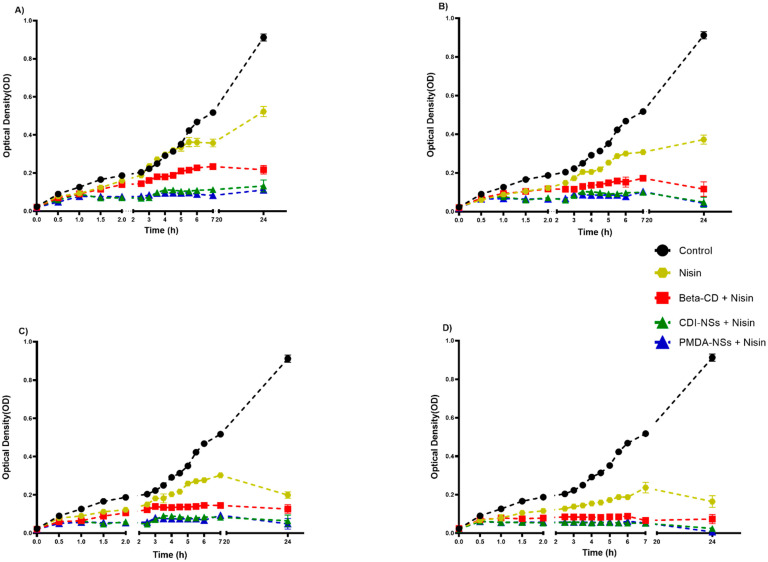
Growth curve of antibacterial activity of nisin-free sample and samples complexed in formulations against *S. aureus*, compared with control and measured by optical density (600 nm) as a function of time: (**A**) 625, (**B**) 1250, (**C**) 2500, and (**D**) 5000 µg/mL. All data are expressed as mean ± standard deviation (*n* = 3). The cited concentrations refer to final nisin concentration; see Section 2.3.1.

BHI broth was used to evaluate bacterial growth at determined time intervals. According to the growth curves, the nisin-free sample could not effectively delay the bacterial growth rate of *E. coli*, even at the highest concentration. However, *S. aureus* strain growth showed a decline with the nisin-free sample, only at 2500 and 5000 µg/mL, whereas the results illustrated a remarkable decrease in bacterial growth, for both strains to the lowest extent at the determined time intervals, when they were exposed to nisin encapsulated with the PMDA and CDI, while the nisin-free sample did not show effective antibacterial activities at mentioned concentrations (Figure 3 and Figure 4). In addition, a considerable bacterial inactivation at concentrations 2500 and 5000 µg/mL of nisin loaded with β-CD against both strains was observed. The main mechanism effect of nisin against bacteria occurs through direct contact of nisin with the bacteria [48,60,61]. It has been reported that CDI-NSs as stable nanosponges formulations are promising carriers for antibacterial protein and prevent depletion of calcium in antibiotic-associated hypocalcemia conditions [62].

Interestingly, drug-free CDI-NSs indicated strong growth inhibition against *E. coli* strains after 24 h incubation at 10,000 and 20,000 µg/mL (Figure S2), whereas PMDA and CDI-NSs showed this inactivation against *S. aureus* (Figure S3) at the last concentration. In contrast, the β-CD sample alone illustrated an inhibitory effect only at 20,000 µg/mL against *E. coli* and *S. aureus*, and it did not show a considerable inhibitory effect for other concentrations, in agreement with the results of agar well diffusion (Figure S1). Furthermore, results of formula size illustrated that the CDI-NS sample had a size of 164.3 nm, which was smaller than other sizes. A strong inhibitory effect of CDI-NS formulation against *E. coli* may be related to its smaller size, as it has been reported that smaller nanoparticles have better bacterial inhibition since they have the highest level of contact with bacteria, so they can easily reach the cytoplasmic content of bacteria [27]; this fact may suggest a higher encapsulation of essential component of bacteria, which would increase the efficacy of the material. The results of this study exhibited antibacterial activities corresponding to rises in both concentration and time.

#### 3.2.3. MIC and MBC

Table 1 shows the actual representative MIC values for *S. aureus* and *E. coli* exposed to nisin Z and to combined forms with PMDA, CDI, and β-CD. The results emphasized that the antibacterial activity of nisin, when combined with the selected formulations, was strongly increased such that the nisin-free sample did not show an inhibitory effect against *E. coli*, even at the highest concentration, but in complex forms with both PMDA and CDI, the formulations decreased to 625 µg/mL. It is worthy to note that, as previously explained, the nisin-free form showed a better inhibitory effect against Gram-positive strains. For this reason, nisin had the MIC value against *S. aureus* at the highest concentration, even though this parameter was strikingly decreased to the lowest concentration for nisin after being combined with NSs. The cell wall of Gram-positive strains is wider than that of Gram-negative; therefore, the antibacterial difference results of our formulations in complex with nisin and nisin-free forms against both bacteria can be elucidated based on this fact. This hypothesis agrees with the cases presented by Kim et al. [63]. Interestingly, NSs alone showed promising MIC values, especially for CDI-NSs against *E. coli*, while β-CD only showed at 20,000 against *E. coli* but not for *S. aureus* (Table 2). Although this kind of potentially bacterial inhibitory effect of poly-cross-likened β-CDs free of antimicrobial agents has been reported to kill both *E. coli and S. aureus* in wastewater treatment [32] and prevent the *Mycobacterium tuberculosis* infection [31], the main difference between these studies and the present report is the different types of cross-linkers used.

To determine the minimum bactericidal concentrations (MBCs), 50 µL of each incubated broth aliquots containing a nisin-free sample and sampled complexed with formulations, as well as drug-free formulations that had shown the MIC values against both pathogens, were taken and plated on MHA plates and incubated for 24 h. The results indicated that the bactericidal effect for nisin was not recognized in both strains, while fortified bactericidal activity was revealed for nisin complexed with CDI in the way that the MBC value for both strains was determined at 625 µg/mL. Nisin encapsulated with PMDA also exhibited a valuable bactericidal effect for both strains, compared with nisin loaded on β-CD (Table 1). In addition, drug-free formulations other than CDI and PMDA at the last concentration and only for *E. coli* strains did not show MBC value, while the β-CD-free sample showed no effect on either strain (Table 2).

#### 3.2.4. Capacity of Bacteria to Use β-CD and Nanosponges as Carbon Sources

**Figure 5:**
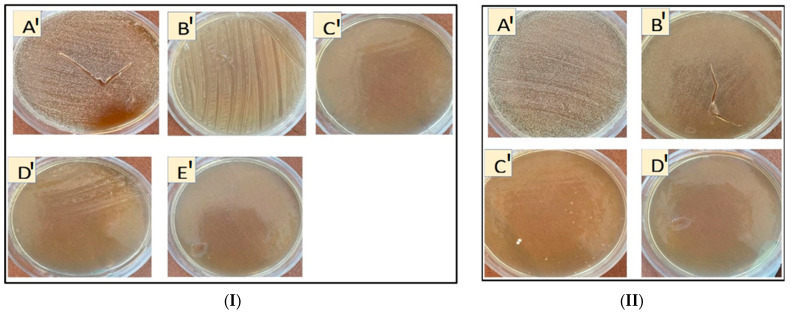
(**I**) Growth of *E. coli* on the surface of minimal agar medium mixed with β-CD and nanosponges as carbon sources: (**A’**) β-CD 20,000 µg/mL; (**B’**,**C’**) PMDA-NS 10,000, 20,000 µg/mL, respectively; (**D’**,**E’**), CDI-NSs 5000, 10,000 µg/mL, respectively; (**II**) growth of *S. aureus* on the surface of minimal agar medium mixed with β-CD and nanosponges as carbon sources: (**A’**) β-CD 5000 µg/mL; (**B’**) CDI-NSs 20,000 µg/mL; (**C’**,**D’**) PMDA-NSs 10,000, 20,000 µg/mL, respectively.

Bacteria have different enzymes; relevant to the present study is amylase [64], which may lead to the breakdown of cyclodextrins as sources of carbon to reduce their antimicrobial activity. Therefore, we used a minimal agar medium free of carbon to evaluate this hypothesis. Interestingly, the results showed that both bacteria grew on plates containing β-CD even at the highest concentrations, which may confirm the hypothesis that β-CD could be used as a carbon source for bacterial growth, while NSs at different concentrations were difficult to digest by the preformed biofilm of *E. coli* or *S. aureus* (Figure 5I,II), in comparison with β-CD alone. Therefore, it can be concluded the NSs are not so suitable as β-CDs to use as carbon sources, likely due to their different 3D structure, which would make degradation by amylases difficult [65].

As a consequence, they were more effective to carry different antimicrobials. Taken together, these results show that PMDA-NSs and CDI-NSs presented interesting antimicrobial activities against Gram-positive and Gram-negative bacteria. The influence of comrade interaction among β-CD monomer and NSs formulations with microorganisms was seen to delay bacterial growth, and in some cases, they could induce the complete inhibition to both strains [66], illustrating a possible mechanism for this inhibition in combination with the delivery of some antimicrobial agents.

#### 3.2.5. Total Viable Counts (TVCs)

One of the most common tests for studies regarding meat quality and shelf-life assessment to investigate meat freshness is total viable counts (TVCs) [64,67]. The antibacterial effects of the nisin-free sample and samples complexed with CDI, PMDA-NSs, and β-CD were defined by the changes in TVCs during storage. Moreover, as a digestive enzyme, pepsin was used to contact with mentioned formulations and nisin-free form to define the protective effect of NSs, changing the antibacterial activities of the nisin-free sample and samples using selected formulations in cooked chicken meat. Table 3 shows the TVC results for cooked chicken meat samples when treated by nisin Z and by nisin encapsulated in various formulations. The initial TVC of the control group was 3.6 log CFU/mL on day 0, while for nisin-free, it was 3.08 CFU/mL, whereas nisin in formulations and in contact with pepsin showed less inhibitory behavior on bacterial growth on day 0. However, over time, at each time point of detection, the effect of nisin and complexes decreased, i.e., the presence of the materials was very insignificant during the first 3, 7, and 14 days, especially in nisin-Z-loaded CDI-NSs. After 30 days, statistical differences in TVCs started to decrease, perhaps due to the degradation of the materials by biotic or abiotic conditions. Statistical differences were observed between the group treated with nisin-free and samples treated with nisin complexed with CDI-NSs (*p* < 0.001) and PMDA-NSs (*p* < 0.01), whereas the nisin sample loaded on β-CD (*p* < 0.05) showed less effect, compared with other formulations.

**Figure 6:**
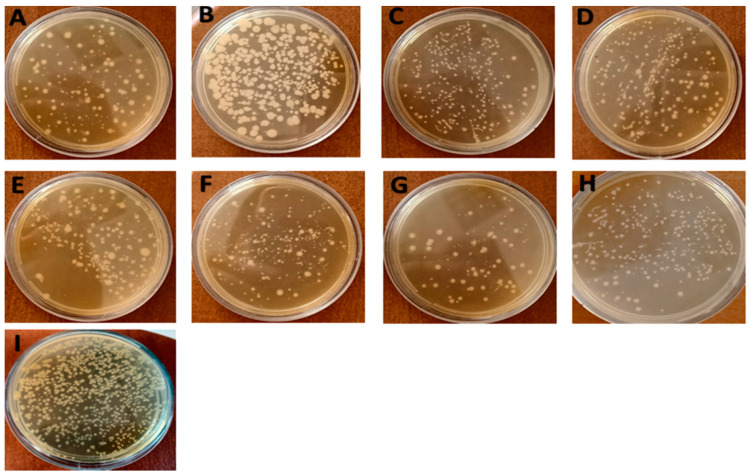
Total viable counts in cooked chicken meat during storage at 30 °C on agar medium: (**A**) 0.05% nisin; (**B**) 0.05% nisin and 0.15% pepsin; (**C**) 0.05% nisin and 0.1% β-CD; (**D**) 0.05% nisin, 0.1% β-CD, and 0.15% pepsin; (**E**) 0.05% nisin and 0.1% PMDA-NSs; (**F**) 0.05% nisin, 0.1% PMDA-NS, and 0.15% pepsin; (**G**) 0.05% nisin and 0.1% CDI-NSs; (**H**) 0.05% nisin, 0.1%, CDI-NS, and 0.15% pepsin (**I**) distilled water (control).

This significant inhibitory effect of nisin Z in formulations might be attributed to the increase in properties such as solubility and stability, as well as the prolonged release of nisin from formulations that remained in samples, showing statistically significant differences during the 30 days of storage time. In comparison, samples treated with nisin in the presence of pepsin showed higher microbial counts (Figure 6), which is similar to the findings of other studies [68,69]. Interestingly, the microbial counts of the nisin-free sample and samples complexed with formulations after contact with pepsin were increased, even though this increase was considerably more for nisin-free than that for complexed samples in NSs and β-CD (*p* < 0.05), especially CDI-NSs (*p* < 0.001). Altogether, the marked reduction in TVCs of chicken meat treated with nisin complexed with the NSs and β-CD, especially NSs, as well as the better antibacterial effect of nisin when encapsulated in formulations in presence of pepsin, might be attributed to the protective effects of NSs and β-CD on nisin, as our previous study indicated the protective effect of PMDA and CDI-NSs for nisin when contacted with pepsin through evaluation of Tricine–SDS–PAGE electrophoresis [33]. In their study, Li et al. showed that nisin loaded on β-CD could delay the breakdown of nisin and increase its stability in the presence of trypsin [42]. The results of the present study indicated that the combination of nisin with NSs and β-CD could act as an effective sanitizer for microbial decontamination in cooked chicken meat. Another study illustrated the inclusion of β-CD complex inhibited the growth of aerobic microflora and reduced survival of *L. monocytogenes* in packaged freshly cut onions [57]. The conclusions drawn in previous studies indicate that nisin activity as an antimicrobial agent in active food packaging can be increased by loading on cellulose nanocrystals [70], chitosan microcapsules [71], chitosan nanoparticles, and chitosan–carrageenan nanocapsule. Indeed, the results revealed a strong antibacterial activity of nisin complexed with NSs against both strains, whereas nisin loaded on β-CD did not show any effect, compared with NS formulations, which may result from further entrapment of any essential biomolecule in the structure of NSs.

It should be noted that various experiments in the present study showed that nisin loaded on PMDA-NSs and the sample free of drug carrier was more effective against Gram-positive bacteria, while nisin complexed with CDI-NSs and its carrier had a higher inhibitory effect against Gram-negative growth; these results might be justified by the different compositions of membranes and the nature of Gram-positive and negative bacteria [56], as well as the different sizes and zeta-potential values of NSs, as previously reported [27,30,31,32].

## 4. Conclusions

In summary, the antimicrobial activity of nisin complexed with PMDA, CDI-NSs, and β-CD was investigated in various antimicrobial experiments such as agar well diffusion, turbid-metric, and MTT assay. The combination of nisin inside PMDA and CDI-NSs illustrated a significant improvement in the quality of cooked chicken meat during 30 days storage, in terms of microbial spoilage (TVC). The results of the release of nisin from the formulations helped to understand their interactions, and the experiments of agar well diffusion and microdilution BHI broth exhibited significantly higher antimicrobial activity of nisin complexed with PMDA-NS and CDI-NS formulations against *E. coli* and *S. aureus*, which corresponded to rises in both concentration and time, whereas less effectiveness was seen when nisin was loaded on β-CD at the same concentrations. Interestingly, drug-free CD-NSs showed antimicrobial effects, as revealed by CDI-NSs and PMDA-NSs showing maximum growth inhibition against *E. coli* and *S. aureus*, respectively, while β-CD alone did not show a considerable effect, compared with NSs.

Finally, the results of this study led us to conclude that PMDA and CDI-NSs have potential antimicrobial activities themselves, as well as the ability to increase nisin stability and antimicrobial activity, which is one of the most common attributes of this food preservative agent and suggests its potential application in the food industry. These results support the idea of a novel antimicrobial formulation based on CD-NS alone or in complexes with some antimicrobials.

## Figures and Tables

**Table 1:** MIC and MBC values for nisin-free sample and samples loaded on selected formulations. ND: not determined.

	MIC (µg/mL)				MBC (µg/mL)			
	Nisin	PMDA + Nisin	CDI + Nisin	β-CD + Nisin	Nisin	PMDA + Nisin	CDI + Nisin	β-CD + Nisin
*E. coli*	ND	625	625	1250	ND	1250	625	2500
*S. aureus*	5000	625	625	1250	ND	1250	625	2500

**Table 2:** MIC and MBC values for drug-free formulations. ND: not determined.

	MIC (µg/mL)			MBC (µg/mL)		
	PMDA-NSs	CDI-NSs	β-CD	PMDA-NSs	CDI-NSs	β-CD
*E. coli*	10,000	5000	20,000	20,000	20,000	ND
*S. aureus*	20,000	ND	ND	ND	ND	ND

**Table 3:** Total viable counts in cooked chicken meat during storage at 30 °C: (C), distilled water; (N), 0.05% nisin; (N–P), 0.05% nisin and 0.15% pepsin; (N-β-CD), 0.05% nisin and 0.1% β-CD; (N-β-CD-P), 0.05% nisin, 0.1% β-CD, and 0.15% pepsin; (PMDA-N), 0.05% nisin and 0.1% PMDA-NSs; (PMDA-N-P), 0.05% nisin, 0.1% PMDA-NS, and 0.15% pepsin; (CDI-N), 0.05% nisin and 0.1% CDI-NSs; (CDI-N-P), 0.05% nisin, 0.1%, CDI-NS, and 0.15% pepsin. Mean values in the same column with the different lowercase letter are significantly different (* *p* < 0.05, ** *p* < 0.01, *** *p* < 0.001, **** *p* < 0.0001 for nisin vs. CD-N, CDI-N and PMDA-N. ^#^ *p* < 0.05, ^##^ *p* < 0.01, ^###^ *p* < 0.001, ns, not significant for N-P vs. CD-N-P, CDI-N-P, and PMDA-N-P). Data are presented as Mean (log CFU/mL) ± SD (*n* = 3).

TVC (log ^CFU/mL^)					
Treatments	Storage Time (d)				
	0	3	7	14	30
C	3.6 ± 0.04	4.8 ± 0.09	5.86 ± 0.05	7.05 ± 0.06	8.8 ± 0.03
N	3.08 ± 0.02	4.43 ± 0.04	5.28 ± 0.05	6.63 ± 0.04	8.5 ± 0.06
N-P	3.45± 0.05	4.72 ± 0.01	5.35 ± 0.04	6.85± 0.07	8.71 ± 0.01
β-CD-N	3.38 ± 0.03	4.30 ± 0.02 *	5.17 ± 0.06 *	6.57 ± 0.02 ^ns^	8.2 ± 0.07 *
β-CD-N-P	3.55 ± 0.02	4.64 ± 0.03 ^#^	5.20 ± 0.02 ^#^	6.70 ± 0.05 ^#^	8.38 ± 0.06 ^#^
CDI-N	3.20 ± 0.06	3.9 ± 0.04 **	4.81 ± 0.04 ***	5.54 ± 0.05 ****	7.88 ± 0.04 ***
CDI-N-P	3.34 ± 0.03	4.18 ± 0.04 ^###^	4.94 ± 0.06 ^##^	5.98 ± 0.03 ^###^	8.07 ± 0.02 ^###^
PMDA-N	3.24 ± 0.03	4.24 ± 0.05 *	5.04 ± 0.05 **	6.33 ± 0.04 **	8.08 ± 0.05 **
PMDA-N-P	3.52 ± 0.05	4.45 ± 0.04 ^##^	5.15 ± 0.03 ^#^	6.54 ± 0.06 ^##^	8.25 ± 0.05 ^##^

## Data Availability

Not applicable.

## Supplementary Materials

The following are available online at https://www.mdpi.com/article/10.3390/pharmaceutics14030685/s1, Figure S1: The inhibitory activity (appearance) of CDI-NSs, PMDA-NSs and β-CD free of drugs. It was evaluated by measuring the growth inhibition zones at different concentration against *E. coli* (A) and *S. aureus* (B) in well agar-diffusion assay; Figure S2: Growth curve of antibacterial activity of drug free formulations against *E. coli* compared to control measured by optical density (600 nm) as a function of time; Figure S3: Growth curve of antibacterial activity of drug free formulations against *E. coli* compared to control measured by optical density (600 nm) as a function of time.
