# Supplementary material for: Cyclodextrin-Based Nanosponges as Perse Antimicrobial Agents Increase the Activity of Natural Antimicrobial Peptide Nisin
Source: Pharmaceutics. 2022 Mar 21;14(3):685. doi: 10.3390/pharmaceutics14030685 (PMC8950107; doi:10.3390/pharmaceutics14030685)
Supplement: Supplementary file 1 [file pharmaceutics-14-00685-s001.zip › pharmaceutics-1612055-supplementary.pdf]

pharmaceutics

MDPI

Supplementary Materials: Cyclodextrin-Based Nanosponges as Perse Antimicrobial Agents Increase the Activity of Natural Antimicrobial Peptide Nisin

Yousef Khazaei Monfared, Mohammad Mahmoudian, Gjylije Hoti, Fabrizio Caldera, José Manuel López Nicolás, Parvin Zakeri-Milani, Adrián Matencio, and Francesco Trotta

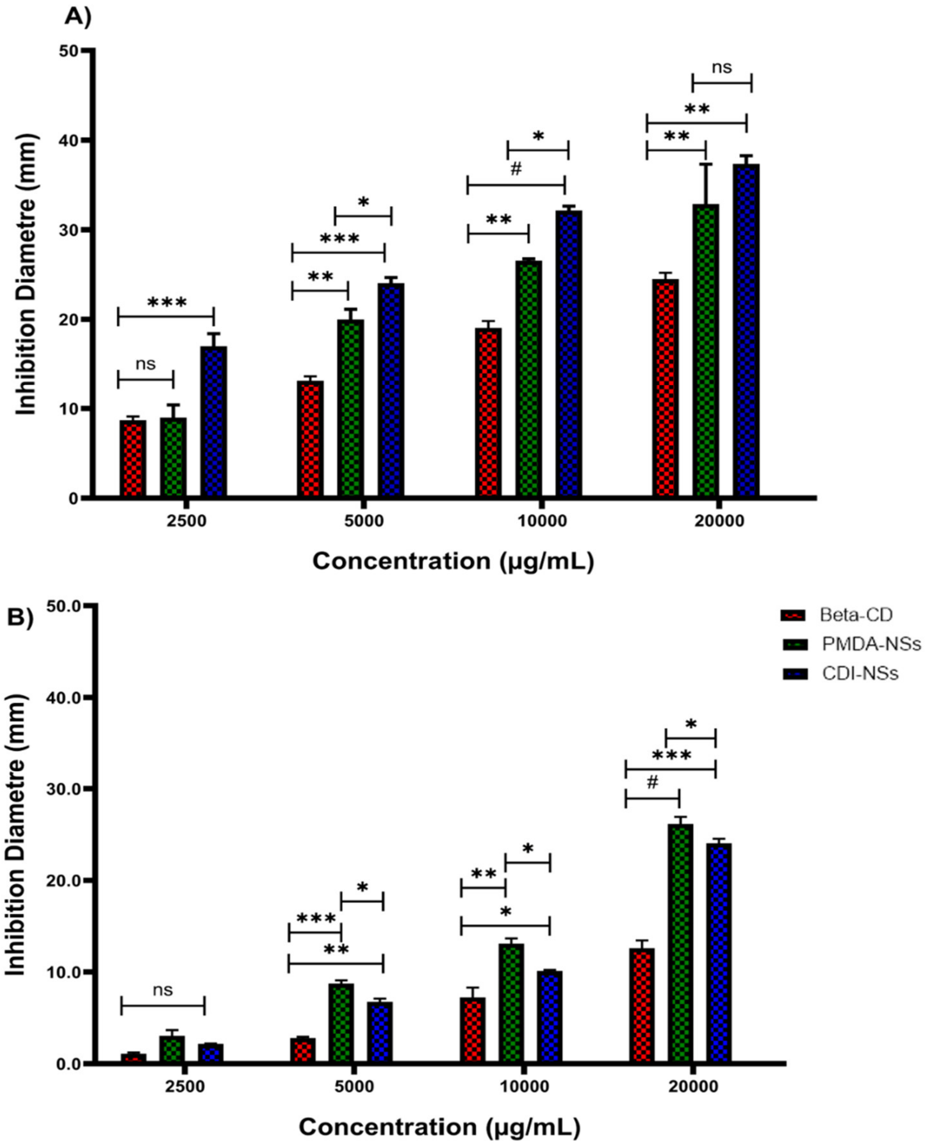

Figure S1. The inhibitory activity (appearance) of CDI-NSs, PMDA-NSs and β-CD free of drugs. It was evaluated by measuring the growth inhibition zones at different concentration against *E.coli* (A) and *S.aureus* (B) in well agar-diffusion assay. **P<0.05, **P< 0.01, ***P< 0.001, #P <0.0001* and ns (Not Significant).

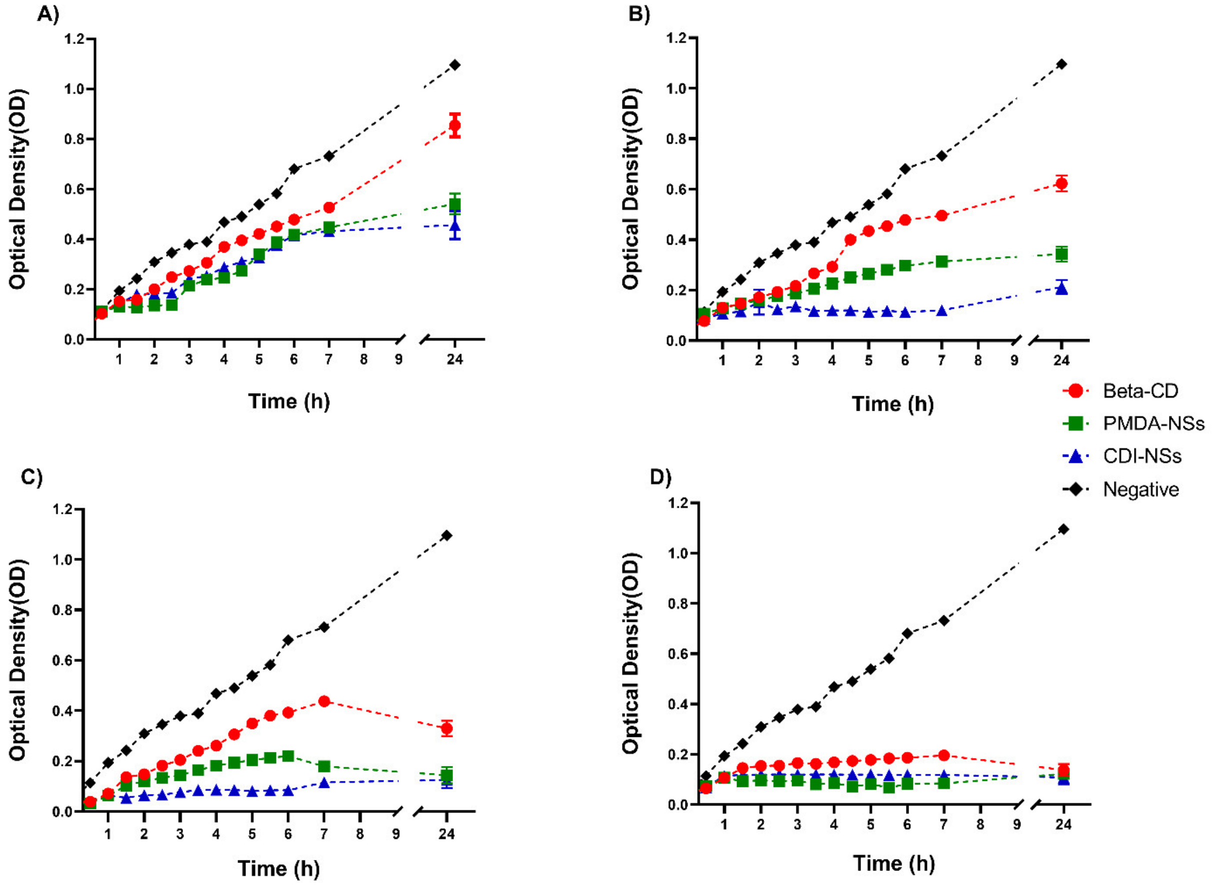

Figure S2. Growth curve of antibacterial activity of drug free formulations against *E. coli* compared to control measured by optical density (600 nm) as a function of time. A: 2500, B: 5000, C: 10000 and D: 20000 µg/mL. All data are expressed as mean ± standard deviation (n = 3).

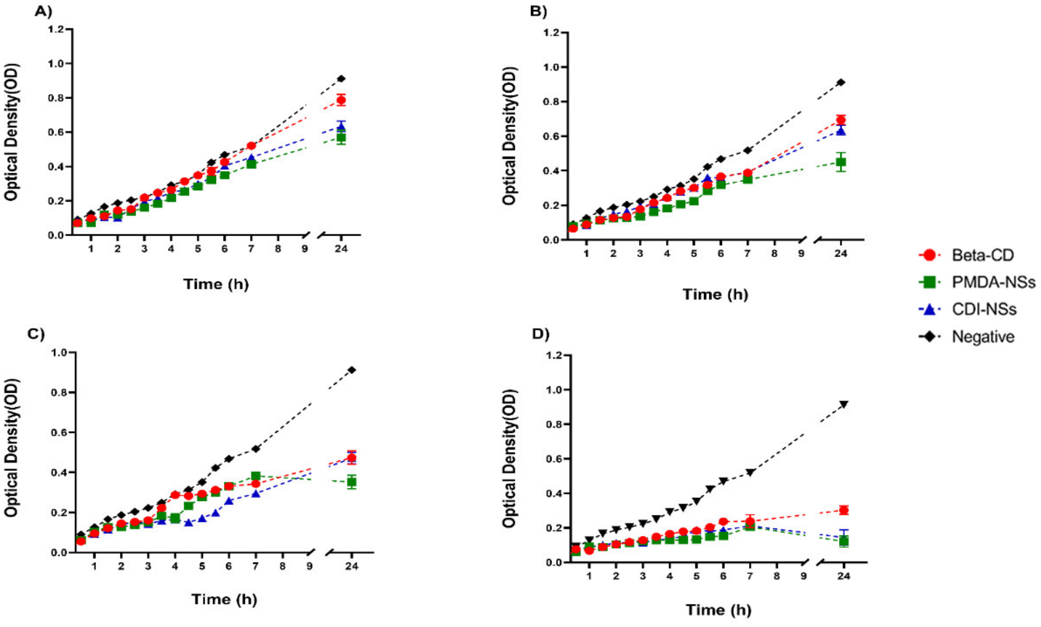

Figure S3. Growth curve of antibacterial activity of drug free formulations against *E. coli* compared to control measured by optical density (600 nm) as a function of time. A: 2500, B: 5000, C: 10000 and D: 20000 µg/mL. All data are expressed as mean ± standard deviation (n = 3).
